# Decision-making and action selection in insects: inspiration from vertebrate-based theories

**DOI:** 10.3389/fnbeh.2015.00216

**Published:** 2015-08-18

**Authors:** Andrew B. Barron, Kevin N. Gurney, Lianne F. S. Meah, Eleni Vasilaki, James A. R. Marshall

**Affiliations:** ^1^Department of Biological Sciences, Macquarie UniversityNorth Ryde, NSW, Australia; ^2^Department of Psychology, The University of SheffieldSheffield, UK; ^3^Department of Computer Science, The University of SheffieldSheffield, UK

**Keywords:** mushroom body, protocerebral calycal tract, leaky competing accumulator model, cross inhibition, parallel inhibition, mutual inhibition, basal ganglia, lateral protocerebrum

## Abstract

Effective decision-making, one of the most crucial functions of the brain, entails the analysis of sensory information and the selection of appropriate behavior in response to stimuli. Here, we consider the current state of knowledge on the mechanisms of decision-making and action selection in the insect brain, with emphasis on the olfactory processing system. Theoretical and computational models of decision-making emphasize the importance of using inhibitory connections to couple evidence-accumulating pathways; this coupling allows for effective discrimination between competing alternatives and thus enables a decision maker to reach a stable unitary decision. Theory also shows that the coupling of pathways can be implemented using a variety of different mechanisms and vastly improves the performance of decision-making systems. The vertebrate basal ganglia appear to resolve stable action selection by being a point of convergence for multiple excitatory and inhibitory inputs such that only one possible response is selected and all other alternatives are suppressed. Similar principles appear to operate within the insect brain. The insect lateral protocerebrum (LP) serves as a point of convergence for multiple excitatory and inhibitory channels of olfactory information to effect stable decision and action selection, at least for olfactory information. The LP is a rather understudied region of the insect brain, yet this premotor region may be key to effective resolution of action section. We argue that it may be beneficial to use models developed to explore the operation of the vertebrate brain as inspiration when considering action selection in the invertebrate domain. Such an approach may facilitate the proposal of new hypotheses and furthermore frame experimental studies for how decision-making and action selection might be achieved in insects.

## Introduction

Decision-making involves analysis and classification of information, and selection of the most appropriate response, which often incorporates reference to memory of what has been learned previously. As a consequence, decision-making involves interaction between many brain systems: sensory, sensory processing, learning and memory, and premotor and motor systems. Effective decision-making is core to the stable operation of any behavioral system, and it is clear that insects are capable of very rapid decision-making (in the order of 10 s of milliseconds; Krofczik et al., 2008; Strube-Bloss et al., 2012a) despite the complexity of the process.

Examining how biological decision-making systems work is an area of extremely active research that combines perspectives from computational biology, machine learning and artificial intelligence with neurobiological analyses of diverse organisms. As mentioned above, decision-making can be divided into the process of information analysis and classification and the process of selection of appropriate responses (action selection). Computationally these two processes are related since they both involve mechanisms for the resolution of competing possibilities to one outcome. Biologically it is increasingly clear that the two processes are not separate, but rather interact and feedback to support a stable outcome.

Our objective here is to consider how effective decision-making might be achieved by the insect brain. By necessity, much of our discussion considers the insect olfactory processing pathway since this is by far the best understood sensory system in insects. We consider how olfactory information might be processed and weighted to contribute to a behavioral choice. First we review the anatomy and operation of the insect brain and the olfactory pathway. We then consider various decision-making mechanisms proposed by biologically-inspired models of decision-making, and neurobiological analyses of the vertebrate basal ganglia (a key region for decision and action selection in the mammalian brain; Chevalier and Deniau, 1990; Redgrave et al., 1999; Bogacz and Gurney, 2007). These provide background for a discussion of how decision-making and action selection might operate within the insect brain. Models of decision-making systems emphasize the need for convergence of evidence-accumulating pathways and the importance of inhibitory elements of the system to drive the resolution of a single outcome from among the possible alternatives. These features of system organization are common to both insect and vertebrate brains. For example, both insect and vertebrate decision-making systems involve convergence of multiple evidence-accumulating pathways that collectively operate a selective relaxation of global feedforward inhibition to enable a unitary decision. We discuss why these similarities might exist.

## The Insect Brain

A major advantage of the insect systems for neuroscientists and modelers is their relative simplicity. In comparison to mammalian brains, insect brains have a simpler anatomy, are far smaller and contain far fewer neurons. The major regions of neuropil and the major tracts connecting them are known for much of the insect brain. The olfactory system is by far the best understood sensory processing system of the insect brain, largely because the key insect models for learning studies (honey bee *Apis mellifera* and *Drosophila*) very readily learn olfactory stimuli in robust lab paradigms. As a result it has been possible to construct a conceptual model of the function of the insect brain that runs from primary olfactory sensory processing to selection of motor output (Galizia, 2014).

**Figure 1:**
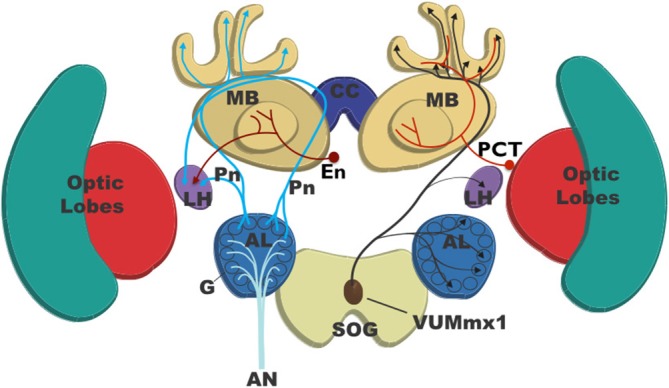
**Basic anatomy of the honey bee brain showing the major pathways involved in odor classification and olfactory learning.** Olfactory receptor neurons send information to the brain via the antennal nerve (AN). These neurons form synapses within the glomeruli of the antennal lobes (ALs) onto local interneurons (not shown) and projection neurons (PN). There are two types of PN: one class projects to both the input region (calyces) of the mushroom bodies (MB) and the lateral horn (LH) and is excitatory. The second class projects to the LH only and is inhibitory. Extrinsic neurons (EN) project from the output of the MB (lobes) to the LH and are inhibitory. Recurrent feedback neurons of the protocerebral-calycal tract (PCT) run from the MB lobes and are inhibitory of both the EN and the MB. During olfactory learning of sugar reward the neuron VUMmx1 neuron (brown) is activated by sugar and releases the neuromodulator octopamine into the LH, AL, and MB, which is believed to contribute to learning-related adjustments in the strength of synaptic connections that enhance appetitive responses to the odor. VUMmx1 is bilaterally symmetrical, but in this figure only the right side is shown.

Significant progress has been made by combining inferences made across different insect systems where this is appropriate, to gain a systems overview of the olfactory processing pathway (Galizia, 2014). The insects are, however, an enormously diverse group: the major orders have evolved independently for at least 300 million years (Grimaldi and Engel, 2005). Insect species can differ enormously in brain size and relative size of different regions of neuropil (Søvik et al., 2015). It is not the intention of this approach (or this section) to imply that all details of brain organization are common to all orders, however basic structural elements of the olfactory pathway are highly conserved across insect orders (Figure 1).

Odors are perceived by antennal olfactory receptor neurons that project to glomeruli within the antennal lobe (AL). Each glomerulus is a region of synaptic contact between axons of olfactory receptor neurons expressing the same olfactory receptors, local neurons (LN) connecting glomeruli, and projection neurons (PN) which convey the odor signal to the mushroom bodies (MB) and/or the lateral horn (LH). The AL is the region of primary olfactory processing where enhancement and sharpening of the odor signal occurs (Galizia and Rössler, 2010; Wilson, 2013; Galizia, 2014). Processing involving LN within the AL sharpens and optimizes the gain of the odor signal (Galizia, 2014). Some of the LN form a network of inhibitory connections between glomeruli such that glomeruli with overlapping response profiles seem to be reciprocally inhibitory of each other (Linster et al., 2005; Galizia, 2014). This appears to be a cross-inhibitory structure, perhaps analogous to the lateral inhibition within retinal processing (Goldstein, 2007). Lateral inhibition in the retina enhances perception of edges (Goldstein, 2007), and similarly the cross-inhibitory network within the AL may sharpen perception of complex odor mixtures increasing the across-glomerular differences in activity and partially decorrelating AL responses to similar odor mixtures (Linster et al., 2005; Galizia, 2014).

The processed odor signal is coded as a specific spatiotemporal pattern of activity across the PN population (Joerges et al., 1997; Galizia and Menzel, 2001; Menzel and Giurfa, 2001). PNs transfer the processed odor signal from the AL to other brain regions, as parallel channels of olfactory information to both the LH and the MB (Kirschner et al., 2006; Brill et al., 2013; Galizia, 2014; Figure 1). In both flies and bees a subset of PNs synapse with multiple glomeruli, and most of these project to the LH and other regions within the lateral protocerebrum (LP). These multiglomerular PN are GABAergic and inhibitory (Kirschner et al., 2006; Liang et al., 2013; Parnas et al., 2013; Galizia, 2014). In both flies and bees a further subset of PNs, the uniglomerular PN, each receive input from a single glomerulus only and most of these project to both the LH and the MB calyx (Fernandez et al., 2009; Denker et al., 2010; Galizia, 2014; in honey bees via two separate tracts; Kirschner et al., 2006; Brill et al., 2013). The uniglomerular PN are excitatory (Fernandez et al., 2009; Denker et al., 2010; Galizia, 2014).

The uniglomerular PNs project to the calyx (input region) of the MB. The odor-evoked signal is there coded as a pattern of activity across the interneurons of the MB: the Kenyon cells (KC). A very small proportion of KC react to any given odor, and different odors are represented by distinct patterns of KC activation (Perez-Orive et al., 2002; Szyszka et al., 2005; Ito et al., 2008; Turner et al., 2008). Processing within the mushroom body (MB) enhances odor identification and classification (Galizia, 2014). The output regions of the KC form the lobes of the MB (Figure 1): there KC connect with extrinsic neurons (EN) that link the MB to the LH, one of the premotor regions of the LP (Rybak and Menzel, 1993). Odor evoked patterns of activity in the EN and LH are believed to shape and select the specific behavioral response to the odor (Strube-Bloss et al., 2012b; Galizia, 2014), as we discuss in “Mechanisms of Decision-Making in the Insect Olfactory Learning Pathway” Section.

### Modeling Information Processing in the Insect Brain

This simple architecture for sensory processing has been an inspiration for modelers, whose theoretical considerations of how information might be efficiently processed within the insect system have helped frame hypotheses for new neurobiological investigations.

**Figure 2:**
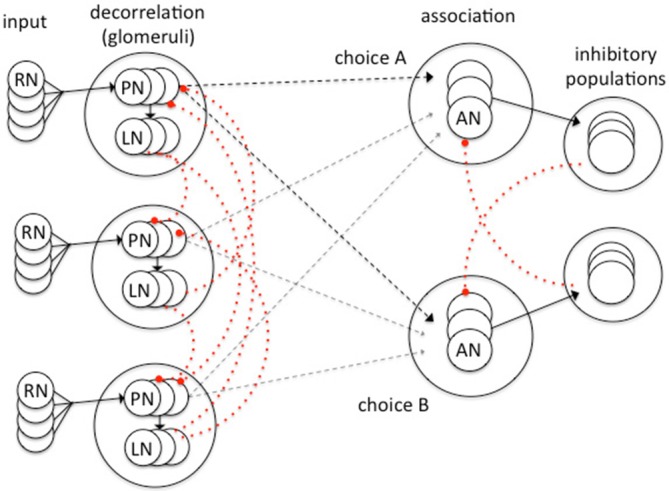
**A model spiking neural network for the classification of multivariate data developed by Schmuker et al. (2014)**. Inhibitory elements in the model (dotted red) are critical for effective system operation. Sensory input from virtual receptor neurons (RN) project to PN that in turn activate local inhibitory neurons (LN) which effect lateral inhibition of PN with similar input properties. This sharpens the sensory input and partially decorrelates the PN responses to similar inputs. This network structure was inspired by processing known to occur in the glomeruli of the insect AL (Galizia, 2014; Schmuker et al., 2014). The output of the decorrelation layer projects to association neurons (ANs) which are grouped in as many populations as there are classes in the dataset. Each population in the association layer corresponds to a choice. Each AN population projects onto associated populations of inhibitory neurons. The strong cross inhibition between AN populations induces a soft winner-take-all behavior in the association layer resulting in a stable unitary choice. Excitatory connections in black, inhibitory connections in dotted red. Plastic connection strengths (adapted by a Hebbian process) shown by dashed lines. Adapted from Schmuker et al. (2014) with permission.

Schmuker et al. (2014) recently created a spiking neural network model inspired by the insect olfactory system (Figure 2) and tested the capacity of the model to correctly classify multivariate data (in this case a four dimensional data set describing features of the petals of different species of iris flowers). Their model structure has three processing layers inspired by an abstraction of the insect olfactory pathway (Figure 2): an input layer (olfactory receptor neurons in the antennae) a decorrelation layer that increases the dissimilarity between similar signals (imagining the glomeruli of the AL and the LN connections between them) and an association layer in which supervised learning occurs (imagining the role of the MB; Schmuker et al., 2014). Their simple model proves to be highly effective in correctly classifying stimuli following a short training period of supervised learning. It is able to output a decision in less than 100 ms of simulated time demonstrating a performance speed that matches that of insects (Schmuker et al., 2014). This high level of performance is achieved by key inhibitory connections within the neural network. Lateral inhibition between neurons of the decorrelation layer enhances differences between similar inputs. It transforms the overlapping receptive fields of virtual receptors neurons into local and specific representations of the input space. Similar processing occurs between the glomeruli of the insect AL (Galizia, 2014). Cross inhibition between neuron populations of the association layer (Figure 2) enables the network to rapidly reach a decision by a soft winner-takes-all behavior since the most active choice inhibits any alternative choices.

**Figure 3:**
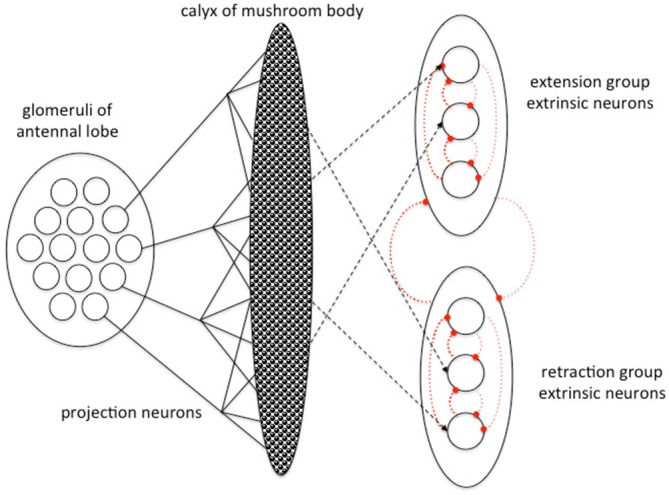
**Abstract model of information processing in the insect olfactory pathway developed by Bazhenov et al. (2013).** PN from the AL convey odor information to the calyx of the MB. MB neurons connect with EN that organize different forms of motor response (here proboscis extension and retraction). Excitatory connections in black, inhibitory connections in dotted red, plastic connection strengths shown by dashed lines. The different classes of EN are connected via cross inhibition to resolve a unitary outcome. Adapted from Bazhenov et al. (2013) with permission.

Schmuker et al. (2014) model explored the efficiency of a simple spiking neural network model as a classifier of multivariate data. Their model structure was inspired by the insect olfactory pathway, but did not intend to explain the function of the insect brain. Bazhenov et al. (2013) however developed a simple mathematical model of how successful odor classification, learning and decision-making might occur within an idealized insect olfactory learning pathway, or any similar neural network (Figure 3). They modeled the pathway simply as two serial connection matrices: the first representing the PN connecting the AL and the MB and the second representing the EN connecting the MB and LH. They considered the experimental case of proboscis extension response conditioning, which is a widely used associative conditioning paradigm for honey bees and *Drosophila* in which restrained insects associate odor with appetitive or aversive gustatory stimuli and learn to extend or withhold their proboscis (Kuwabara, 1957; Bitterman et al., 1983; Giurfa and Sandoz, 2012). Their model (Bazhenov et al., 2013) considers these two possible behavioral responses only, and imagines EN to be activational of either proboscis extension or retraction. These two populations of EN are modeled as cross-inhibitory such that neurons of the extension and retraction populations inhibit each other, and neurons within a functional population are mutually inhibitory as well (Figure 3). With this system EN receiving a lot of excitatory input would fire, and also silence other EN. If active EN belonged mostly to the extension population the insect would extend the proboscis, otherwise the proboscis remains withheld.

In the model (Bazhenov et al., 2013) connections between KC and EN are assumed to be plastic and modified by three processes. Appetitive reward strengthens connections between active KC and extension group EN while weakening connections with the retraction group EN via the action of neuromodulators on KC output synapses. No reward or an aversive stimulus does the opposite: weakening connections between active KC and extension group EN while strengthening connections with the retraction group EN. Operating on a slower timescale, in the absence of any appetitive reward connection strengths between KC and the extension group EN decay over time, while connection strengths between KC and the retraction group EN increase by a simple Hebbian process.

Bazhenov et al.’s (2013) proposes a simple and elegant mechanism for the decision process. During training with a specific odor paired with an appetitive reward connection strength between the KC responding to the odor and extension group EN strengthen, while connections to retraction group EN weaken. Once a threshold activation of extension group EN is reached the retraction group EN are shut down by cross inhibition and the insect extends its proboscis in response to the learned rewarded odor. This simple model captures many of the known features of insect PER learning: rapid decision, rapid learning and a non-linear step change in behavioral response to a learned odor that is relatively stable thereafter.

The models proposed by Bazhenov et al. (2013) and Schmuker et al. (2014), however, imagine a very simple action selection problem. In both their models there was only one appropriate action for each presented stimulus, and the model learned to make the appropriate selection in response to different stimuli. For a behaving animal the problem of action selection is far more complex. There are multiple possible responses to any given set of stimuli, and the most appropriate response (in terms of furthering the animal’s survival or reproduction) may vary according to the time of day, the animal’s condition or physiological state, or the broader environmental state (such as the season). Understanding the neurobiology of action selection is a complex challenge. Below we consider how the problem of effective action selection has been approached by computational neuroscientists.

## Models of Decision-Making and Action Selection

**Figure 4:**
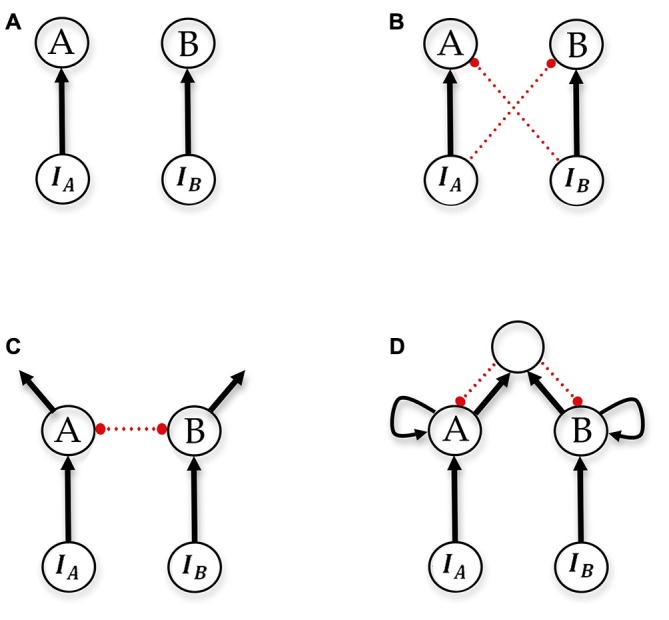
**Schematics of accumulator models.** **(A)** race (Vickers, 1970), **(B)** feed-forward inhibition (Ditterich et al., 2003), **(C)** leaky competing accumulators (LCAs; Usher and McClelland, 2001) and **(D)** pooled inhibition (Wang, 2002). Neuron populations A and B are decision populations integrating evidence over time, *I*_A_ and *I*_B_ are the respective sensory populations. Either population A or population B must reach an activity threshold in order for a decision to be made. In the pooled inhibition model, there is a shared pool of inhibitory interneurons. Arrows denote excitatory connections, small circles and dotted red lines denote inhibitory connections. Adapted from Bogacz et al. (2006) and Marshall et al. (2012).

In computational models describing perceptual decision-making in vertebrates, various proposals have been made as to how evidence-accumulating pathways should be connected (Figure 4). The simplest and oldest accumulator model is now referred to as the “race model”, in which evidence accumulating pathways are completely independent and unconnected, and “race” to reach a variable decision threshold in order to precipitate a decision (Vickers, 1970). Subsequent proposals couple the evidence-accumulating pathways in various ways, such as cross inhibition with decay (known as the leaky competing accumulator (LCA) model), feed-forward inhibition, and pooled inhibition (Figure 4). The effect of these couplings is to implement a winner-take-all mechanism, by ensuring that as integrated evidence in one pathway (corresponding to activation in an integrator population) increases, it suppresses the activity of other pathways. While these dynamics and their benefits are intuitive, further support for the importance of coupling evidence pathways has been presented by formal analyses of the statistical tests the proposed models can implement. Bogacz and colleagues (Bogacz et al., 2006; Bogacz, 2007) analyzed linear versions of the LCA model, the feed-forward inhibition model, and the pooled-inhibition model, and showed that all three can, under appropriate parameterizations, be approximately reduced to the same one-dimensional model of decision-making, the drift-diffusion model (DDM). This is particularly important because the DDM, arising in the psychological literature (Ratcliff, 1978, 1981, 1988, 2002) has been the most successful in explaining reaction time and accuracy data in diverse psychophysical experiments, and because the DDM corresponds with the sequential probability ratio test (SPRT) as evidence accumulation moves from discrete to continuous. The SPRT is a sequential statistical test that optimally compromises between expected accuracy of a decision, and the expected number of samples required to reach it. The DDM integrates the difference in available evidence, and thus without coupling of evidence pathways no model can implement or approximate it. For this reason the race model is provably sub-optimal, since evidence accumulating pathways are completely uncoupled. The analyses mentioned above assume that the appropriate performance criterion for decision-makers is accuracy based; i.e., decision-makers should seek to optimally compromise between expected speed of decision and accuracy of decisions, with these the only two factors influencing when a decision is reached. In more ecological decision scenarios, in which the expected value of decisions should be optimized rather than expected decision accuracy (Pirrone et al., 2014), value-sensitive decision-making mechanisms also make use of cross-inhibitory connections (Seeley et al., 2012; Pais et al., 2013).

### Models of Learning

As described above implementation of decision-making models requires particular wiring of brain areas representing stimuli and action selection. The analyses described in the previous section are valid for “proficient-phase” decision-making, in which the value of stimuli have been learned or evolutionarily hard-wired, but these models cannot account for how the value of simuli can be learned or modified by experience. Clearly this is a key aspect of decision making for most animals, and various models have been proposed for how learning might be achieved by brains.

When it comes to mechanisms for learning the value of stimuli, brain self-organization models suggest that the interaction of Hebbian learning rules (where the synapse of co-active neurons is strengthened) which link stimuli to actions, and winner-take-all-like (or lateral connectivity) architectures that impose the selection of a unique action, are required. Though mainly inspired by the mammalian brain, a correlation rule of the form

(1) weight ​change ~ f(pre) f(post)

where f(pre) and f(post) are functions of presynaptic and postsynaptic activities (usually firing frequencies) could be expected to be quite a general solution in animal brains, because of its simplicity and fundamental properties. Such a two-factor rule is the basis of classical models of unsupervised (Oja, 1982; Kohonen, 1989) and developmental learning (von der Malsburg, 1973; Bienenstock et al., 1982) that describe how connection strengths in a circuit might change as a consequence of their current interactions.

This simple two-factor formulation is insufficient for the formation of stimuli-action relations when a feedback signal is presented (either rewarding e.g., food or aversive) in learning goal-oriented behavior. It is the current belief that rewarding situations are represented in the brain by changes in the concentration of neuromodulators that are available to and shared by large neural populations. In some vertebrate brain areas, dopamine has been identified as a candidate signaling unexpectedly rewarding situations (Schultz, 1997, 2001, 2007). It is then a reasonable step to extend the Hebbian rule by adding a third factor representing the reward available to the system:

(2) weight ​change ~ f(R) f(pre) f(post)

where f(R) is a quantity that depends on the neuromodulator concentration related to the level of reward that the selected action brought to the system. The consequence of this formulation is that the expected weight change will only be made conditional on the presence of the appropriate neuromodulator, resulting in learning behavior only of specific rewarding (or punishing) events. It is also possible to extend this form of synaptic modification to capture precise spike-timing activity rather than firing frequencies (Vasilaki et al., 2009; Gurney et al., 2015). Models of the form described above have also been used to explain how learning of reward and punishment might occur within the insect MB where the neuromodulators octopamine and dopamine are involved in the reinforcement of reward and punishment learning respectively (Schwaerzel et al., 2003; Vergoz et al., 2007; Bazhenov et al., 2013). For architectures involving rules of this form and lateral connectivity that lead to unique action selection, see for instance (Richmond et al., 2011).

## Biological Mechanisms of Choice and Decision-Making in Vertebrates and Invertebrates

The models discussed above provide a theoretical perspective on how choice and decisions might be efficiently achieved by neural networks, and how animals might learn new behavioral responses to stimuli. The models emphasize that inhibitory elements are key to the successful operation of the system. In this section we review what is known of decision-making processes and action selection in the vertebrate basal ganglia and the insect olfactory learning pathway. While these two examples are derived from different phyla, it is not clear if they represent completely independent evolutions of decision-making systems. Strausfeld and Hirth (2013) have argued the possibility of a deep homology between the vertebrate basal ganglia and protocerebral structures of the insect brain. Even if this is correct it remains the case that insect and vertebrate lineages have been evolving independently for an estimated billion years, during which time both groups have independently evolved more complex sensory and motor systems and new forms of behavior. Any commonalities in mechanisms of decision-making found in both insects and vertebrates might therefore represent general operating principles for decision-making in evolved neural networks.

### Mechanisms of Action Selection in the Vertebrate Basal Ganglia

**Figure 5:**
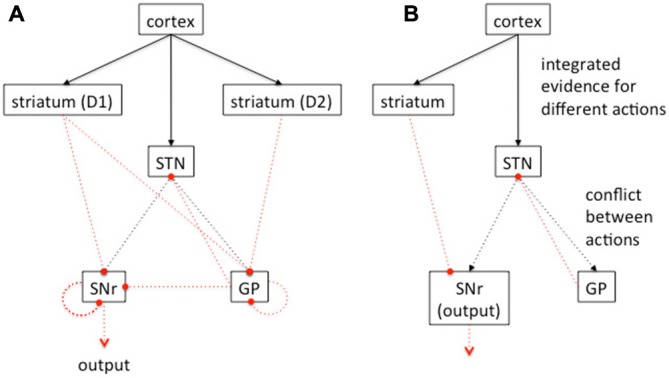
**Vertebrate basal ganglia circuitry. (A)** Simplified schematic of major connections between the basal ganglia. Excitatory connections in black, inhibitory connections in dotted red. Focussed projections solid lines, diffuse projections dashed lines. Cortical input reaches both the striatum and the subthalamic nucleus (STN). The striatum is divided into two populations of PN, expressing the D1 or D2 type dopamine receptors respectively. Neurons in the D1 population send their principal projections to the substantia nigra pars reticulata (SNr). In primates, these striatal projections also go to globus pallidus (GP) internal segment, and in rats, to the entopeduncular nucleus (not shown here for clarity) with SNr subsuming the generic role of “output nuclei”. Neurons in the D2 striatal population send their principal projections to the GP in rats, and the external segment of the GP in primates. Both SNr and GP receive input from the STN; the GP (or GP external segment) reciprocates that projection. Both GP and SNr contain local intra-nucleus connectivity. Constant inhibitory output from SNr reaches widespread targets in the thalamus and brainstem. **(B)** Further abstraction of the basal ganglia connections to show an architecture capable of theoretically performing a MSPRT analysis. Connection styles as in **(A)** Adapted from Bogacz (2007) and Bogacz and Gurney (2007) with permission.

The vertebrate solution to the problem of action selection is believed to be critically dependent on the basal ganglia (Figure 5), a set of subcortical nuclei which have a long evolutionary lineage, and which are highly conserved (Stephenson-Jones et al., 2012).

The basal ganglia receive inputs from all over the brain including wide areas of the cortex (excluding primary sensory areas) and many subcortical nuclei (McHaffie et al., 2005). According to the action selection hypothesis, these inputs comprise “action requests” whose overall activity represents the urgency or “salience” of the action (Redgrave et al., 1999; Gurney et al., 2001). The action requests are then processed through “action channels” running through basal ganglia, which are subject to competitive processing, thereby causing selection of those channels with the highest salience. The effect of the competition must then be made manifest at the originating structures making the action requests. The mechanism for effecting this is selective *release* of inhibition (Chevalier and Deniau, 1990). That is, the basal ganglia output nuclei normally supply tonic (continuous) inhibition to their targets, and release this inhibition on representations of the selected actions therein. Thus, the result of internal competition in basal ganglia is a *decrease* in the activity of the selected channels at the level of the output nuclei (and possibly an increase in activity in non-selected channels).

Basal ganglia have a plethora of neuronal mechanisms to support the competitive processing described above. At the systems level (across the entire basal ganglia), there are the possible components of a feedforward, off-center on-surround network meaning activity in the focal action channel is inhibited and activity in alternative channels enhanced (Mink and Thach, 1993; Gurney et al., 2001). The striatum supplies a channel-wise, focused (off-center) projection to the output nuclei, while the subthalamic nucleus (STN) supplies a diffuse projection (on-surround); see Figure 5. Channels with large salience will depress activity in their basal ganglia outputs, while simultaneously forcing outputs on competing channels high. Note the polarity of this network is opposite to that which might be used in many selection circuits such as those described in the insect brain, because as described above the “winning” channel must have its output depressed, not enhanced.

A computational model of basal ganglia which invoked this off-center, on surround circuit was described in Gurney et al. (2001). As well as showing basic selection properties using this circuit, the model also suggested a role for the globus pallidus (GP) and, in particular, its inhibitory connections to STN (Figure 5). Thus, the GP is in a recurrent loop with the STN and, the more excitation the GP receives from STN, the stronger its effect back at STN. This results in an “automatic gain control” reminiscent of such systems in engineering signal processing. As such, it keeps the overall level of diffuse excitation from STN at the output nuclei, at the correct level for competitive selection, independent of the number of action channels taking part. Another (complementary) interpretation of this feedback circuit from GP to STN is suggested by the mapping of the basal ganglia onto the statistical decision making algorithm—the multihypothesis sequential probability ratio test (MSPRT; Bogacz and Gurney, 2007; Lepora and Gurney, 2012). There, in a Bayesian scheme, the STN and GP combine to produce exactly the required combination of likelihoods for the calculation of the log posterior probabilities for deciding a course of action. One heuristic interpretation of this computation is that it combines a simple “race” model (disinhibition via striatum to output nuclei) with “conflict resolution” (in STN and GP).

Turning to the wider anatomical scheme, basal ganglia receive input from cortical and subcortical areas developing nascent action representations (McHaffie et al., 2005). Focusing on the cortical influence, the cortical representations are in recurrent excitatory circuits with thalamus to which basal ganglia project. Thus, by selectively releasing inhibition at the thalamus, basal ganglia allow activity in the cortico-thalamic loops to increase, thereby allowing the associated action channel to be expressed.

It has been shown how this circuit can work (Humphries and Gurney, 2002) and how it can manifest behavior using embodiment in a small mobile robot (Prescott et al., 2006). One of the key features for the success of the latter model was the way in which the salience for an action was built from non-linear combinations of sensory input. Such pre-processing of sensory information could be a feature of the olfactory guidance of behavior in insects where olfactory glomeruli in the AL perform complex transforms of their input before sending projections to LP (Galizia, 2014).

The basal ganglia support, not only the feedforward architecture for competition described above, but several other possible competitive mechanisms. GP and the output nuclei both contain intrinsic inhibitory collaterals, which could be the basis of competition (Deniau et al., 1982; Park et al., 1982). A model incorporating these connections (Gurney et al., 2004) showed that they could indeed improve the selection capability but, interestingly, only when found together—collaterals in the output nuclei alone hindered selection. Thus, the general application of lateral or cross inhibition is, counter-intuitively not always an aid to selection.

The motif of lateral inhibition is also supported within the striatum. Here there is a complex GABAergic microcircuit (Bolam and Bennett, 1995), which could support competitive processing by inhibition between populations of striatal PN supporting different action channels (Alexander and Wickens, 1993; Fukai and Tanaka, 1997). The inhibition between these so-called medium spiny neurons (MSNs) is generally not “mutual”; a neuron MSN1 may inhibit MSN2 but this does not imply MSN2 inhibits MSN1 (Tepper et al., 2004). Nevertheless there is a possibility of selection processing using such connectivity (Tomkins et al., 2014). There is also a possible indirect inhibitory interaction between channels supported by (excitatory) cortical projections to inhibitory striatal interneurons, which, in turn, innervate MSNs (Tepper et al., 2004). GABAergic connectivity is also observed within the output nuclei (Deniau et al., 1982) that could support competitive processes. Notice that, within the striatum, “winning” channels need to have their activity enhanced not suppressed, while within the output nuclei the opposite prevails (as noted above). Nevertheless, inhibition can support both outcomes as it provides a general signal contrast enhancement—its effects are transparent to the meaning of the signals themselves.

The striatum also provides mechanisms for selection at the neuronal level. MSNs require substantial coherent input to fire (Wilson and Kawaguchi, 1996). This could act to filter low salience inputs, which may be considered as noise rather than genuine action requests. In addition the transient dynamics of MSNs under changes in input can act to enhance selection, and these transients are a property of the neurons rather than the circuits in which they are embedded (Tomkins et al., 2014).

The emphasis on local inhibitory collaterals as a mechanism for selection is a hallmark of the models of basal ganglia by Frank and co-workers (Frank et al., 2004, 2007; Frank, 2005). These models contain collaterals in cortex, as well as the main nuclei of the basal ganglia and it is this combination of multiple loci of cross inhibition, which facilitates selection. The more recent models also emphasize the role of STN as suppressing action “by effectively raising decision thresholds in the face of conflict” (Frank et al., 2007), a notion consistent with that in the heuristic description of MSPRT.

### Mechanisms of Decision-Making in the Insect Olfactory Learning Pathway

In “The Insect Brain” Section we introduced the insect olfactory learning pathway, here we propose how decision-making and action selection might occur within this pathway. Figure 1 illustrates the key brain regions and connecting neural tracts involved in the insect olfactory learning pathway using the honey bee as an example. Table 1 summarizes the functions of the major neural populations and how activity in these populations changes during olfactory learning.

**Table 1:** **Summary of the major regions of neuropil and tracts involved in the insect olfactory learning pathway, and how they change activity during associative conditioning with reward and punishment**.

Neural population	Inputs	Outputs	Proposed function	Activity changes with reward conditioning	Activity changes when conditioning with no reward or punishment
Uniglomerular projection neurons (PN)	Each neuron receives input from a single glomerulus within the antennal lobe (AL)	MB calyx and lateral horn (LH)	Excitatory. Conveys information relevant to odor identity to input regions of the MB and to the LH	In honey bees discriminant conditioning with one rewarded and one unrewarded odor may shift the representation of both odors in the PN population in such a way to improve the separation and discrimination between the two odors (Fernandez et al., 2009; Denker et al., 2010). But see Parnas et al. (2013) which presents no evidence for any shift in coding in PN population with aversive stimulation in *Drosophila*.
Multiglomerular PN	Multiple glomeruli within the AL	Ventrolateral protocerebral neurons (Liang et al., 2013) in LH	Inhibitory. GABAergic. Respond to summed activity across glomeruli. Provide gain control to olfactory signal to LH to sharpen odor discrimination (Parnas et al., 2013). Contribute to selection of behavioral response by selective inhibition of different classes of odor signal e.g., food vs. sex pheromone: parallel inhibition (Liang et al., 2013).	Presently unknown	Presently unknown

Kenyon cells (KC)	Olfactory input from PN within the Calyx of the MB	EN and protocerebral tract (PCT) neurons within lobes of the MB	Excitatory. Olfactory information sparse-coded within KC population to enhance odor classification	Consolidate and strengthen KC responses to learned odor, recruit additional KC responses (Faber and Menzel, 2001; Szyszka et al., 2008)	Weaken KC responses to learned odor (Szyszka et al., 2008)
Extrinsic neurons (EN)	Lobes of the MB	LH and other regions of the LP	Presumed inhibitory. Contributes to selection of behavioral response by differing levels of inhibition of premotor regions.	Identified EN PE1 decreases activity (Okada et al., 2007). A small proportion of other EN also reduce activity, a smaller proportion increase activity (Okada et al., 2007)	Identified EN PE1 did not change firing with the absence of a sucrose reward (Okada et al., 2007)
Mushroom body (MB) feedback neurons	PN within calyces of MB (Ganeshina and Menzel, 2001) and KC within lobes of MB (Rybak and Menzel, 1993)	KC and PNs within calyx of MB (Ganeshina and Menzel, 2001) and EN within lobes of MB (Okada et al., 2007)	Inhibitory, GABAergic. Provides tonic inhibitory input to KC, and a degree of inhibition of EN	At the level of individual neurons reduced activity has been recorded (Grünewald, 1999b; Okada et al., 2007), but as a population average increased activity to CS+ observed (Haehnel and Menzel, 2010)	As a population average, decreased activity to unrewarded odor observed (Haehnel and Menzel, 2010)

As discussed above, both theory and the example of the mammalian basal ganglia emphasize that decision-making mechanisms rely on a convergence of evidence accumulation pathways on a single locus within which the degree of conflict between different outcomes can be determined and resolved. For insects LP may represent such a locus. The LP is premotor, and organizational of different possible motor responses. The LH is a subset of the LP that receives processed olfactory input (Galizia, 2014; Ito et al., 2014) and outputs via ventrolateral protocerebral neurons to other regions of the LP (Liang et al., 2013; Parnas et al., 2013). The LP also receives processed visual information via the central complex and MB (Pfeiffer and Homberg, 2014). The LP is therefore a premotor region of convergence of processed sensory information. Regarding olfactory inputs to the LP, these are concentrated to the LH, which receives two direct channels of olfactory information. An excitatory input preserves the cross-glomerular pattern of activity and therefore preserves information on odor identity, and an inhibitory input conveys summed activity across glomeruli. The LH also receives an indirect channel of olfactory information via the MB, which we discuss later. A model emerging from recent studies with *Drosophila* proposes that the function of the LH is to transform information on odor identity into the degree to which the odor input is activational of different specific behavioral responses. This is achieved by converting information on odor identity into a measure of valence (here defined as the degree to which a stimulus will elicit a positive or negative behavioral response; Parnas et al., 2013; Fişek and Wilson, 2014; Galizia, 2014) and by channeling information on different fundamental classes of odors (e.g., food odors or sex pheromones) to different specific premotor circuits in other regions of the LP, including the ventrolateral protocerebral area (Liang et al., 2013; Parnas et al., 2013; Galizia, 2014).

**Figure 6:**
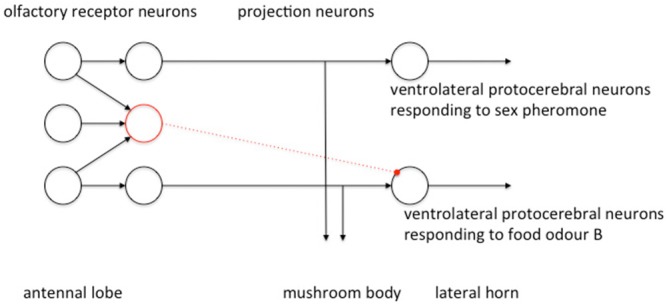
**Parallel organization of excitatory and inhibitory signals in the PN population modified from Liang et al. (2013) with permission.** Excitatory connections in black, inhibitory connections in dotted red. Uniglomerular PN each receive input from a single glomerulus and output excitatory signals in parallel to the LH. The multiglomerular PN sum inputs from multiple glomeruli and appear to inhibit downstream LH neurons in a channel-specific manner. LH neuron responses to food odors are inhibited whereas responses to sex pheromones are not.

Evidence gathered so far suggests that odors that are either chemically similar or that activate a similar behavioral response activate a similar region of the LH (Parnas et al., 2013; Fişek and Wilson, 2014) and that the LH is spatially organized by valence such that odors of similar valence cause similar spatial patterns of activity in the LH. This is organized by the arrangement of the excitatory uniglomerular PN inputs to the LH (Parnas et al., 2013). The inhibitory multiglomerular PN inputs provide a form of gain control to the olfactory signal and enhance dissimilarities of similar odors to refine odor resolution (Parnas et al., 2013). The inhibitory multiglomerular PN input is also channel-specific (Liang et al., 2013). In flies the multiglomerular PN synapse with ventrolateral protocerebral neurons within the LH, which are then activational of other regions of the LP (Liang et al., 2013). Multiglomerular PN are activated by food odors and exert a general inhibition of the responses of downstream ventrolateral protocerebral neurons to at least two food odors, but not to two different sex pheromones (Liang et al., 2013). This has been described as parallel inhibition (Figure 6), in which excitatory and inhibitory PN receive parallel input and send parallel output to the LH (Liang et al., 2013). Functionally this could selectively route different classes of odor information to different circuits within the LP. Liang et al. (2013) speculate that this mechanism of parallel inhibition gates food odor input to activate LH neurons relevant to foraging responses, and sex pheromone input to activate LH neurons relevant to courtship and mating responses, and therefore contributes to a mechanism of action selection.

The valence of an odor and the degree to which it activates the LH is further influenced by an indirect olfactory input to the LH via the MB, and it is via this pathway that valence of an odor can be influenced by learning. Uniglomerular excitatory PN project to the calyces of the MB (as well as directly to the LH) where they synapse with the KC interneurons that form the MB (Figure 2). KC output via the lobes of the MB, and synapse with EN that project to the LH (Figure 1). Odors are sparse-coded within the numerous KC, and the MB has a capacity for fine-scale odor discrimination and classification (Bazhenov et al., 2013; Galizia, 2014). Odor coding within the MB is also plastic and influenced by learning, as a result of the actions of neuromodulators octopamine and dopamine (released in response to reward and punishment) modulating the connection strengths of the output of the KC (Schwaerzel et al., 2003; Burke et al., 2012; Perry and Barron, 2013; Søvik et al., 2015). Training of odors associated with sugar reward changes and enhances the KC population reacting to the odor (Szyszka et al., 2008) and also changes and generally decreases activity in the EN population (Okada et al., 2007) as a consequence of neuromodulators triggered by the rewarding stimulus adjusting synaptic connection weights within the MB (Burke et al., 2012; Perry and Barron, 2013; Søvik et al., 2015). Odor processing via the MB pathway therefore provides a mechanism for experience-dependent adjustment of the valence of an odor stimulus.

The gain of the odor signal within the MB is regulated by recurrent GABAergic inhibitory MB feedback neurons that receive input from PN in the calyces of the MB and from KC in the lobes of the MB, and that output via both the calyces and lobes to PN, KC and EN populations (Rybak and Menzel, 1993; Grünewald, 1999a; Ganeshina and Menzel, 2001; Okada et al., 2007; Liu and Davis, 2009; Haehnel and Menzel, 2010; Hu et al., 2010; Palmer and Harvey, 2014). In both bees and flies the feedback neurons are a small population: in bees they are the approximately 50 neurons of the protocerebral tract (PCT; Mobbs, 1982; Bicker et al., 1985; Grünewald, 1999a), in flies they are the anterior paired lateral neurons (Liu and Davis, 2009). These neurons provide inhibitory feedback to the MB and PN and are important for maintaining sparse coding, and therefore fine-scale odor resolution, within the MB (Perez-Orive et al., 2002; Lei et al., 2013; Palmer and Harvey, 2014), and contribute to learning-related plasticity in the PN, MB and EN populations (Denker et al., 2010; Haehnel and Menzel, 2010; Raccuglia and Mueller, 2014).

Kenyon cells are excitatory of EN (Okada et al., 2007), but the EN are inhibitory of the LH and downstream LP premotor circuits (Rybak and Menzel, 1998; Okada et al., 2007). Activity in one identified and well studied EN PE1 (identifiable from its specific firing properties) is negatively correlated with the probability of a behavioral response (Okada et al., 2007), but the responses of the EN population as a whole are plastic and influenced by experience so that for odors learned to be predictive of reward the amount of activity in the EN population on average reduces and therefore the degree of inhibition of the LH imposed via the MB circuit is selectively relaxed (Rybak and Menzel, 1998; Okada et al., 2007; Haehnel and Menzel, 2010; Galizia, 2014).

To summarize; a general model of the insect olfactory learning pathway is that odor information is processed in parallel via a direct AL—LH connection and an indirect AL—LH connection running via the MB. Odor identity is coded as specific temporo-spatial activity first within the glomeruli of the ALs and then within the uniglomerular PN population. Odor discrimination and classification is enhanced by sparse coding across the more numerous KC population. Excitatory and inhibitory channels converge at the LH where their combined inputs act on premotor neurons projecting to other regions of the LP. Parallel inhibitory inputs carried by the multiglomerular PN to the LH contribute to action selection by gating channels of olfactory information to different classes of behavioral output. The excitatory input direct to the LH from the uniglomerular PN provides a signal of valence of the odor. This valence signal is modulated by learning processes acting via the MB channel, which is usually globally inhibitory of the LH unless the animal has learned an odor has a high valence in which case MB inhibition of the LH is selectively relaxed.

The process of decision-making in the insect olfactory learning pathway is illustrated by how the system changes as an animal learns a specific odor is rewarding and changes its behavior to effect an appetitive response to the odor (Table 1). With training the pattern of activation of both glomeruli and uniglomerular PN shifts to increase the distinction between the rewarded odor and similar odors (Faber et al., 1999; Abel et al., 2001; Galizia and Menzel, 2001; Fernandez et al., 2009; Denker et al., 2010; Smith et al., 2012). Reward conditioning sharpens and enhances KC responses to the odor to enhance odor classification (Szyszka et al., 2008), and as a consequence the population of EN reacting to the odor also changes and on average reduces activity (Okada et al., 2007). This selectively releases neurons within the LH from inhibition via the MB channel and enables activation of a behavioral response (Okada et al., 2007). GABAergic inhibitory signals from the MB feedback neurons are also changed, and this contributes to these plastic changes in the MB, PN and EN populations (Liu and Davis, 2009; Palmer and Harvey, 2014; Raccuglia and Mueller, 2014), and sharpens the signal at these three points in the pathway (Perez-Orive et al., 2002).

The inhibitory elements are clearly key to effective operation of the insect olfactory learning pathway. Inhibitory MB feedback neurons exhibit a general inhibitory tone across the network to optimize gain of the olfactory signal. The inhibitory regulation of the LH by the multiglomerular PN has been described as parallel inhibition to channel specific classes of olfactory information to specific premotor pathways within the LP. The action of the EN on neurons within the LH is inhibitory, with selective relaxation of the degree of inhibition for rewarded odor. Excitatory and inhibitory inputs converge at the LH. The net result of the inhibitory and excitatory odor inputs to the LH for a rewarded odor is a sharpening and increase of the specific odor-evoked excitatory input to the LH via the uniglomerular PN, a channeling of that information toward specific motor responses by the multiglomerular PN and an odor-specific relaxation of inhibition of the LH by the MB input via the EN population.

### System Similarities Between the Mechanisms of Action Selection Within the Vertebrate Basal Ganglia and the Insect Brain

Far more is known about the mechanisms of action selection in the vertebrate brain than is known for the insect brain, but knowledge of the mechanisms in the vertebrate basal ganglia can help frame hypotheses for further investigation of the insect brain. At a high level of abstraction there are some similarities of system organization between the two brains, although the specifics of the systems are very different. In both brains the mechanism of action selection involves a convergence and coupling of multiple evidence accumulating pathways involving both excitatory and inhibitory signaling.

As discussed in the “Mechanisms of Action Selection in the Vertebrate Basal Ganglia” section the action of the basal ganglia can be summarized as combining inputs to release from inhibition the output of the selected behavioral channel and enhance inhibition of other similar but non-selected channels providing a form of off-center : on-surround mechanism for the selection of a specific channel. The polarity of this network is counterintuitive since the BG is globally inhibitory of motor pathways and hence a channel is selected by a selective reduction in inhibition. One mechanism for achieving this is by combining a focussed inhibitory projection from the striatum (off-center) and a diffuse excitatory projection from the STN (on-surround).

We propose the mechanism of action selection in insects can be considered in a similar way (albeit with an opposite polarity) by combining inputs in the LP to provide an on-center: off-surround mechanism for action selection. The LP combines a focussed excitatory projection from the uniglomerular PN (on-center) with a diffuse inhibitory projection from the multiglomerular PN (off-surround) and a selective relaxation of inhibition from the MB pathway (effectively also on-center : off-surround). The LP is almost certainly a point for convergence of processed visual information from the visual lobes, MB and central complex also, and rather like the BG may be a common path for convergence of processed information as part of action selection and decision-making.

Bogacz and Gurney have argued that the basal ganglia achieves decisions by the implementation of the MSPRT statistical test (Bogacz, 2007; Bogacz and Gurney, 2007). This is a generalization of the SPRT able to consider multiple hypotheses such that the decision process can be described as competition between multiple alternatives with the evidence for each alternative being computed at each moment to yield a probability for each alternative being correct. A decision is made when the probability for any alternative exceeds a threshold. The thresholds are not fixed, but are functions of the degree of conflict between alternatives such that the more conflict the more stringent the probability threshold becomes (Bogacz, 2007).

The MSPRT approximates an optimal solution to the decision-making problem; Bogacz and Gurney (2007) argue the basal ganglia is capable of these calculations, by combining evidence for each alternative provided by the striatum with the degree of conflict between alternatives computed by the subthalamic nuclei and GP (Bogacz, 2007; Bogacz and Gurney, 2007). This has proved a very useful hypothesis for critiquing basal ganglia function. Can the insect LP be interpreted in a similar way? It would be interesting to consider if the insect olfactory pathway might also be capable of theoretically resolving a MSPRT test, or an equivalent test for optimizing the reward/decision time tradeoff for decisions in which reward obtained is more important than realized decision accuracy (Pirrone et al., 2014).

For the insect brain to be capable of this kind of calculation it would need a capacity to integrate the evidence for making each of all possible choices and separately calculate the sum of the exponents of the evidences for all choices (Bogacz, 2007; Bogacz and Gurney, 2007). A decision can then be made when the evidence for one choice in comparison to the total amount of evidence for all choices exceeds a threshold (Bogacz, 2007; Bogacz and Gurney, 2007). In the insect brain various sensory processing pathways could be theoretically capable of integrating evidence for different possible choices. The insect olfactory pathway involving the AL and MB and converging on the LH, described in detail here, is certainly capable of an evidence accumulation function for the olfactory sense. The larger question is whether the insect brain is capable of summing *all evidence* for all choices. In the vertebrate brain this function is performed by the STN and the GP (Figure 5) as a common path for cortical output. In the insect brain there would need to be a point of convergence and integration of information across all sensory systems. This locus may be the LP. In honey bees the MB is multisensory and capable of fine-scale classification of sensory information and learning of the valence of that information (Menzel, 2001; Menzel and Giurfa, 2001; Galizia, 2014). MBs output to the LH, which also receives inputs from the AL, visual lobes and the central complex, which processes visual, mechanosensory and somatosensory information relevant to space (Pfeiffer and Homberg, 2014; Seelig and Jayaraman, 2015). Anatomical evidence would suggest the LP as a candidate for total evidence summation, and the premotor nature of the LP proposes a possible role for action selection, but presently we know too little about how information channels converge in the insect brain, in the LP or any region, to speculate further on whether an MSPRT type calculation, or equivalent, might be possible in the insect brain.

When exploring this hypothesis it would be interesting to examine the function of the neuromodulators involved in signaling valence (reward and punishment) within the LP. Dopamine in vertebrates, as well as playing a key role in learning, also acts directly to modulate neural excitability in basal ganglia (Onn et al., 2003; Frank, 2005). In this way, it may play a direct role in promoting or impeding action selection. Octopamine in honey bees is strongly linked to reward singaling, and also modulates neural excitability (Weisel-Eichler and Libersat, 1996; Menzel, 2001; Vehovszky et al., 2005). In honey bees during learning of sugar reward octopamine is released at multiple points in the olfactory pathway (MB, AL and LP; Hammer, 1993; Hammer et al., 1993; Hammer and Menzel, 1995), and modulates function and odor coding in the AL and MB (Farooqui et al., 2003), but the specific role in the LP is presently unknown (Søvik et al., 2015). Nevertheless, neuromodulators are hypothesized to contribute to mechanisms of “motivational switching” in the LP (Galizia, 2014). Evidence of such a role for octopamine therein, may help support the thesis that the LP acts to support decision making as the basal ganglia do in vertebrates.

## Conclusion

Effective action selection is a problem faced by even the simplest of organisms (Bray et al., 2007; Bray, 2009) and it is reasonable to assume that one of the key functions of the simple brain of the earliest motile animals must have been to solve the action selection problem. From this perspective it is not surprising that in both vertebrates and invertebrates action selection appears localized to evolutionarily older regions of the brain (Mink and Thach, 1993; Redgrave et al., 1999; Strausfeld, 2012). Some authors have even proposed an ancient evolutionary homology between these regions (Strausfeld and Hirth, 2013), and it is possible that distant common ancestors could have possessed action selection brain structures that evolved into two distinct but related structures, one arthropod and one vertebrate.

Here we have taken a different approach, asking what design patterns have been detected in, and proposed for, vertebrate and invertebrate brain regions involved in learning, decision-making, and action selection. In particular, we have identified the theoretical importance, and empirical prevalence, of coupling of evidence-accumulating pathways, particularly in decision-making and action selection. Theoretical and computational studies have shown that such coupling, which can be implemented using a variety of different mechanisms, greatly improves the performance of decision-making systems. Indeed, the results of a recent computational model inspired by decision-making models of vertebrate brain regions have shown that the removal of inhibitory coupling gives rise to inefficient action selection (Marshall et al., 2015). We believe that modeling approaches developed to explore the operation of vertebrate brain regions such as the basal ganglia can be borrowed and adapted to propose new hypotheses for how action selection might be achieved in insects.

We have reviewed what is known about invertebrate decision-making in light of the more complete experimental and theoretical considerations of the vertebrate basal ganglia and proposed some new hypotheses for the operation of decision and action selection in insects. In this review we have emphasized the role of the LP in action selection in insects, since this is a premotor region within which there is convergence of sensory processing pathways for the well-studied olfactory learning pathway. The LP is to date a relatively poorly understood region of the insect brain, partly because it is anatomically less well defined than sensory processing regions or the MB, partly because it is less accessible than the sensory lobes and partly because in the honey bee literature at least it has historically been seen as not involved in the olfactory learning pathway (Hammer and Menzel, 1998), which may be a misconception (Søvik et al., 2015). We hope this review will inspire new research into the function of this region, and the importance of inhibitory signaling in the decision-making process in the insect brain.

## Conflict of Interest Statement

The authors declare that the research was conducted in the absence of any commercial or financial relationships that could be construed as a potential conflict of interest.

## Abbreviations

AL: antennal lobe
DDM: drift diffusion model
EN: extrinsic neuron
LCA: leaky competing accumulator
LN: local neuron
LH: lateral horn
LP: lateral protocerebrum
MB: mushroom body
MSPRT: multihypothesis sequential probability ratio test
MSN: Medium spiny neuron
PN: projection neuron
SPRT: sequential probability ratio test.
